# Modification of a desktop FFF printer via NIR laser addition for upconversion 3D printing

**DOI:** 10.1016/j.ohx.2024.e00520

**Published:** 2024-03-19

**Authors:** Adilet Zhakeyev, Rohith Devanathan, Jose Marques-Hueso

**Affiliations:** a Institute of Sensors, Signals and Systems, Heriot-Watt University, EH14 4AS, Edinburgh, United Kingdom; b UMDO, Instituto de Ciencia de los Materiales, University of Valencia, Valencia, 46980 Spain; c Applied Physics Department, University of Valencia, Valencia 46980 Spain

**Keywords:** Upconversion 3D printing, NIR laser stereolithography, FFF printer modification

## Abstract

Traditional photopolymer-based 3D printing methods require sequential printing of thin layers, due to short penetration depths of UV or blue light sources used by these techniques. In contrast, upconversion 3D printing circumvents the layer-by-layer limitation by taking advantage of upconversion luminescence processes and the high penetration depths offered by near-infrared (NIR) lasers, allowing for selective crosslinking of voxels at any depth or position within the resin container. The implementation of this technique required the construction of a 3D printer with the ability of focusing the laser on any point of the space. For this, a low-cost fused filament fabrication (FFF) printer was modified by incorporating a 980 nm laser and laser control circuit. The total cost of the parts required for modification was £180. With enhanced penetration depths up to 5.8 cm, this method also allows for printing inside or through existing 3D printed parts. This opens doors for restoration of broken items, in situ bioprinting, 3D-circuitry, and notably, 3D printing inside cavities of a different material, illustrating numerous opportunities for practical applications.

## Graphical abstract

**Figure:**
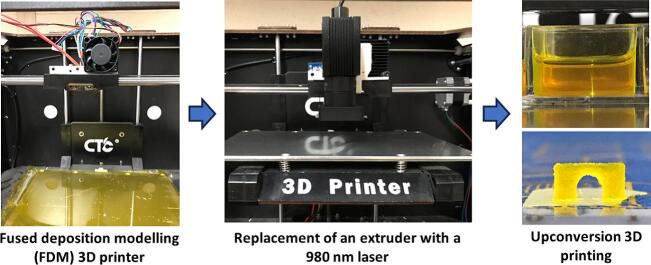

Specifications table.

**Table:** 

Hardware name	Upconversion luminescence 3D printer
Subject area	Engineering and materials science
Hardware type	Mechanical engineering and materials science
Closest commercial analog	Stereolithography 3D Printers – e.g. Form 3 (Formlabs)
Open source license	CC BY 4.0
Cost of hardware	£180
Source file repository	http://doi.org/10.17632/tdh45sybsn.1

## 1 Hardware in context

Photopolymer-based AM techniques such as stereolithography (SLA), digital light processing (DLP), liquid crystal display (LCD) printing and continuous liquid interface production (CLIP) offer high resolution compared to other 3D printers [1], [2]. This group of techniques involve layer-by-layer solidification of a liquid resin via photo-crosslinking under light irradiation [3]. Recently, new strategies have been developed for volumetric 3D printing at a macroscopic scale [4], [5], [6], [7]. For instance, tomographic methods, in which a container with photopolymer resin is rotated, whilst multiple images are projected into the volume of the target material at defined angles [4], [5]. However, these methods mostly utilize ultraviolet (UV) or blue light (400–450 nm) for excitation, which exhibit inherently short penetration depths. Several visible light photoinitiators (up to 700 nm) have been developed in recent years [8], however the one-photon absorption process used for initiation can result in unwanted dose accumulation, as well as producing curing induced by the background light, limiting their use in photopolymer-based 3D printing.

In contrast to the one-photon absorption-based methods, two-photon polymerization (TPP) is a well-established technique that induces crosslinking in the volume rather than on surfaces. TPP utilizes two-photon absorption (TPA), where a molecule absorbs two photons at the same time by using a virtual state and a long wavelength excitation source (∼800 nm) [9], [10]. TPP offers an unbeatable resolution, but it is more suitable for fabrication of nano/microstructures due to the slow printing speeds and limited working distances of the focusing optics. Moreover, due to the TPA process requiring the use of high-intensity femtosecond lasers, the TPP printers are very expensive [11]. Whilst in upconversion luminescence, the sequential absorption of low-energy photons results in subsequent emission of a higher-energy photon. In rare-earth doped phosphors, there are several main upconversion mechanisms: excited state absorption (ESA) [12], energy transfer upconversion (ETU) [13], [14], cooperative sensitization (CS) [15] and photon avalanche [16]. Amongst upconversion processes, ETU is the most efficient one, since it involves at least one order of perturbation less [13], [14]. In lanthanide-based UC phosphors, the probability of transition for a two-photon process can be approximately one million times higher than systems that use virtual energy levels (TPA, second-harmonic generation, etc.), due to real energy levels, which means that the required laser power density is significantly smaller compared to TPP [13]. Hence inexpensive continuous-wave (CW) lasers can be used in upconversion-assisted 3D printing. Since most of the common photopolymer formulations are active in the UV region, ytterbium (Yb^3+^)/thulium (Tm^3+^)-doped phosphors had been recently used for in-volume printing via upconversion [17], [18], [19], [20], [21], [22]. Upon excitation of Yb^3+^ ions (^2^F_5/2_ → ^2^F_7/2_) with 980 nm photons, three successive energy transfers from Yb^3+^ to Tm^3+^ populate ^3^H_5_, ^3^F_2_ and ^1^G_4_ energy levels of Tm^3+^ [23], [24]. The UV and blue emissions of Tm^3+^/Yb^3+^ co-doped phosphors arise from the following transitions: ^1^I_6_ → ^3^H_6_ (∼291 nm); ^1^I_6_ → ^3^F_4_ (∼347 nm); ^1^D_2_ → ^3^H_6_ (∼363 nm); ^1^D_2_ → ^3^F_4_ (∼454 nm) and ^1^G_4_ → ^3^H_6_ (∼475 nm) [25]. The use of NIR light (980 nm) for excitation allows for much higher penetration depths in photopolymer resins [17]. The enhanced penetration depth, coupled with the effective optical nonlinearity, can introduce new functionalities, such as repair of broken parts and restoration of artifacts, embedded electronics, and in situ bioprinting.

**Fig. 1:**
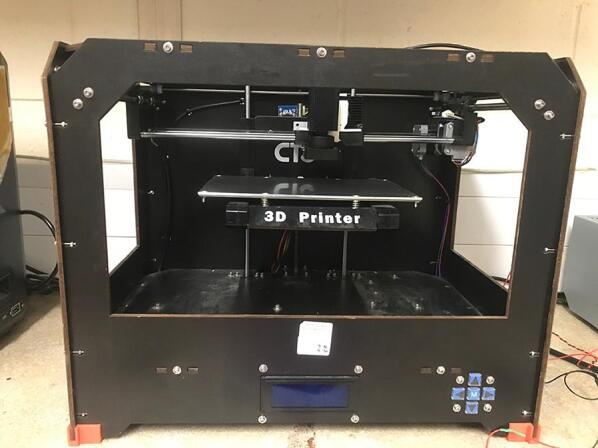
Modified FFF printer, with an integrated 980 nm laser.

In the research described here, a desktop FFF 3D printer is repurposed with a low cost 980 nm NIR laser diode, as shown in Fig. 1. This allows for selective crosslinking of photopolymers at the desired depth achieved using both micron-sized commercial upconversion phosphors and core/shell/shell upconversion nanoparticles [21], [22]. The control of the laser is achieved with a custom circuit, whilst the g-code commands are generated with open-source ReplicatorG software with a modified open-source printer profile. This 3D printer was also used for fabrication of parts/features inside of previously 3D printed parts.

## 2 Hardware description

Commercial SLA, DLP, and LCD 3D printers utilize UV or visible light sources, which limits the existing printer configurations to either ‘top-down’ or ‘bottom-up’ approaches, where a thin layer of resin is confined between the build platform, necessitating the use of a resin re-coating mechanism after the previous layer is cured. The exposure happens always at a 2D surface that is fixed in the space. Whilst TPP, a volumetric microfabrication technique, is prohibitively expensive, with commercial printers, such as microFAB-3D (MicroLight 3D) and Photonic Professional GT2 (Nanoscribe) exceeding $120000 [26]. Since most SLA-based 3D printers are proprietary and are based on ‘top-down’ or ‘bottom-up’ mechanisms, with a constant 2D exposure region, their modification would be challenging. Moreover, open-source versions, such Prusa’s SL1S Speed cost around $2000, and utilize an LCD screen for excitation which would not provide enough power density necessary for upconversion processes. There are open-source DIY SLA configurations, such as the one reported by Andy Rosen, where the components required for X,Y and Z axis movement and control result in a total build costs in the $600–800 range [27], however our upconversion multi-material technique requires that the 2D curing surface is movable in the volume.

In recent years the widespread availability of affordable and open-source desktop FFF printers has enabled the introduction of additional functionalities. For instance, the integration of an additional syringe-based extrusion printhead enabled the use of modified FFF printers in bioprinting [28] and food 3D printing [29] applications. Furthermore, the introduction of UV light source in addition to the syringe-based extrusion printhead had enabled curing of photopolymer resins, liquids, pastes, and gels [30], [31]. In another application, the addition of a xenon lamp to a FFF printer allowed a hybrid printing process for Flash Light Assisted Manufacturing of structural Electronics (FLAME), where the intense pulsed light (IPL) was used to sinter silver nanoparticles [32]. Whilst laser cutting and engraving has been introduced to FFF printers with integrated powerful blue laser (405–445 nm) diodes [33], [34]. Moreover, laser assisted FFF 3D printing had been demonstrated, in order to increase the inter-layer bonding strength of the printed parts [35], [36].

This article is intended to take advantage of the affordability and ease of use of the FFF printers to extend their application to NIR-assisted photopolymer 3D printing, with enhanced penetration depth capability compared to traditional SLA-based 3D printers. Specifically, a clone of an open-source Makerbot Replicator - CTC Bizer (CreateBot, Zhuhai City, China) is outfitted with a low-cost 980 nm NIR laser diode (OFL371, OdicForce Lasers, Surbiton, UK). The laser diode alongside the optical system was attached in place of an extruder via a 3D printed clip-on mount using an open-source CAD design by David Granz [37]. The control of the laser is achieved via a custom-built circuit, which is connected to the FFF printer in place of the extra cooling fan. The designs in the STL format were converted into a G-Code using ReplicatorG software [38] and a modified printer profile [37] to give the commands to successfully 3D print structures via upconversion-assisted crosslinking of photopolymer resin, at substantial depths through the resin (>1 cm).

The widespread availability of FFF printers, which nowadays can be found in university labs as well as hobbyists’ garages, makes them an attractive option for enabling NIR-assisted upconversion photopolymer 3D printing. This will allow researchers to test their synthesized NIR to visible light upconversion materials, such as upconversion nanoparticles (UCNPs) consisting of an inorganic host matrix doped with lanthanide ions [11], in 3D printing applications at low-cost. Moreover, one distinctive feature of this printer is its ability for selective crosslinking at varying depths, which can allow for printing features inside of previously SLA/DLP printed parts, enabling the addition of features consisting of multiple materials and colors. Additionally, despite utilizing moderately viscous resins for the fabrication of demonstrators shown in this work, our printer modification does not require layer re-coating and multiple immersions, which makes it applicable for high viscosity resins. Furthermore, in our prior works we had used this modified printer alongside a formulation containing silver ions for fabrication of conductive metalized parts on different substrates, such as glass and 3D printed polymers via selective electroless copper plating [21], [22].

Broad Applications for Researchers:

1. Reduction of waste: the nonlinearity of upconversion processes and NIR excitation allows for iterative modifications in the design (including printing with a different material for additional functionality), which can be imparted on the previously 3D printed iteration of the design, therefore reducing the number of prototypes.
2. Embedded electronics: with the modification of the photopolymer resin and selective plating, conductive tracks can be plated on previously 3D printed parts.
3. Repair and restoration: the ability to selectively crosslink at varying depths combined with strong penetration depth of NIR light has a potential in applications such as repair of broken parts or aiding restoration of delicate, complex artifacts without creating a replica.
4. 3D printing of highly viscous photopolymers: this technique does not require layer re-coating and build platform immersions.

## 3 Design files summary

**Table:** 

Design file name	File type	Open source license	Location of the file
Design file 1	Figure	CC BY 4.0	http://doi.org/10.17632/tdh45sybsn.1
Design file 2	Video	CC BY 4.0	http://doi.org/10.17632/tdh45sybsn.1

**Design file 1**: schematics of the circuit for laser control.

**Design file 2**: video of the 3D printer during operation (printing under already 3D printed structure).

## 4 Bill of materials summary

**Table:** 

Designator	Component	Number	Cost per unit -currency	Total cost -currency	Source of materials	Material type
Laser housing 1	980 nm laser	1	£29.99	£29.99	OdicForce	Other
Laser housing 2	Heatsink / Laser Diode Housing	1	£12.5	£12.5	OdicForce	Metal
Laser housing 3	M3 screws	2	£0.336	£0.672	RS Components	Metal
Laser optics 1	N-BK7 Plano-Convex Lens, Ø1″, f = 25 mm (LA1951)	2	£20.30	£40.60	Thorlabs	Glass
Laser optics 2	Lens holders (cylindrical)	2	£24.02	£48.04	Thorlabs	Metal
Laser optics 3	Lens holders (square)	1	£15.83	£15.83	Thorlabs	Metal
Laser optics 4	Adapter with External SM1 Threads and Internal SM05 Threads	1	£16.78	£16.78	Thorlabs	Metal
Laser optics 5	Retaining Ring for Ø1″ Lens Tubes and Mounts	2	£3.65	£7.3	Thorlabs	Metal
Laser control 1	MOSFET	1	£0.361	£0.361	RS Components	Electronics
Laser control 2	Laser Driver board	1	£12.99	£12.99	OdicForce	Electronics
Laser control 3	Breadboard	1	£13.84	£13.84	RS Components	Electronics
Laser control 4	Optocoupler	1	£0.28	£0.28	RS Components	Electronics
Laser control 5	470 Ω Resistor	1	£0.18	£0.18	RS Components	Electronics
Laser control 6	2.2 kΩ Resistor	1	£0.037	£0.037	RS Components	Electronics
Laser control 7	Breadboard Jumper Wire Kit	1	£3.74	£3.74	RS Components	Electronics
Laser control 8	Red wire (1 m)	1	£0.244	£0.244	RS Components	Electronics
Laser control 9	Black wire (1 m)	1	£0.249	£0.249	RS Components	Electronics
Laser control 10	5 V AC to DC Adapter	1	£5.99	£5.99	Amazon	Electronics

### 5 Build instructions

Equipment/tools required for assembly include:

• FFF and RepRap-class 3D printer
• Hex key
• Soldering iron
• Bench power supply unit (PSU)

*Preliminary steps:*

**Fig. 2:**
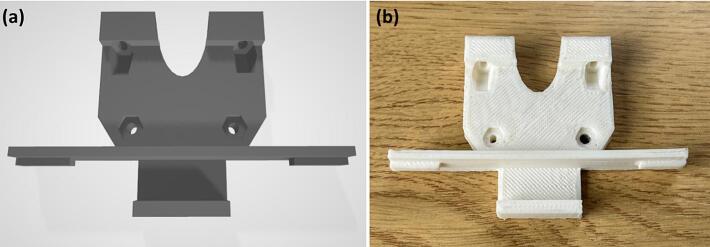
(a) CAD model of the clip-on mount [37]; (b) Clip-on mount 3D printed with ABS filament using Replicator 2X (MakerBot) FFF printer.

**Step 1:** A mechanical mount is required in order to attach the laser to the FFF printer. In this case, an open-source design (CC BY-SA 3.0) of a clip-on mount was 3D printed using ABS polymer, using Replicator 2X (Makerbot), as shown in Fig. 2 (it can also be 3D printed with the same FFF printer to which the laser will be attached). The CAD file of the mount design created by David Granz is available on Thingiverse under the identifier 770,634 [37]. This mount design can be used on CTC Bizer 3D printers, as well as any other Replicator (Makerbot) clones, where it can be installed directly in front of the extruders. Alternative open-source mount designs (CC BY-SA 3.0) are available on Thingiverse**,** however they might require further modifications to the printhead, for instance milling out the middle part between the two extruders [39].

*Laser/optical system assembly:*

**Fig. 3:**
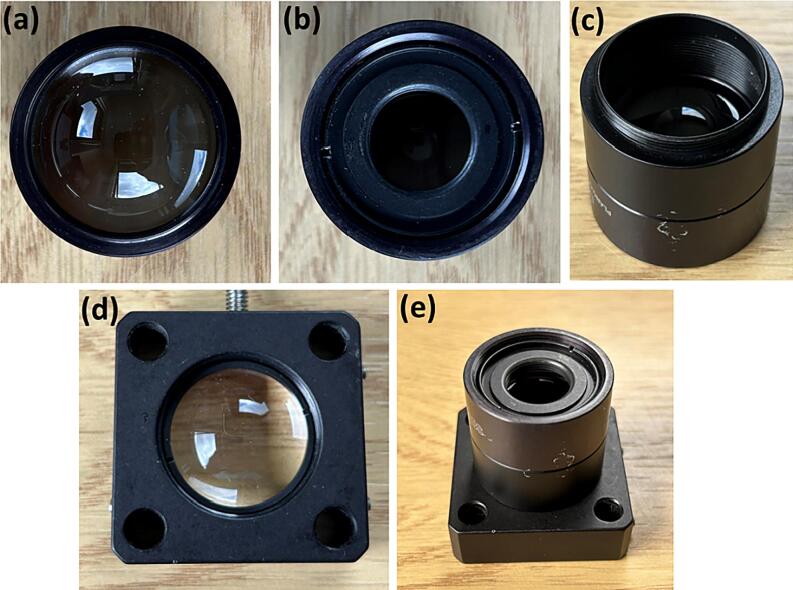
(a-e) Assembly of laser optical components for beam collimation and focusing.

**Step 2:** Insert the plano-convex lens (**Laser optics 1**) all the way down into the cylindrical lens holder (**Laser optics 2**), with the convex part of the lens facing outwards, as shown in Fig. 3 (a).

**Step 3:** Insert the lens adapter (**Laser optics 4**) into the cylindrical lens holder (**Laser optics 2**), as shown in Fig. 3 (b).

**Step 4:** Attach the second cylindrical lens holder (**Laser optics 2**), to the one assembled in **Steps 2**–**3**, as shown in Fig. 3 (c).

**Step 5:** Fix the second plano-convex lens (**Laser optics 1**) inside the square lens holder (**Laser optics 3**), using two retaining rings (**Laser optics 5**), as shown in Fig. 3 (d).

**Step 6:** Attach the parts assembled in **Steps 2**–**4** to the end with the convex part of the lens of the part assembled in **Steps 5**, as shown in Fig. 3 (e).

**Fig. 4:**
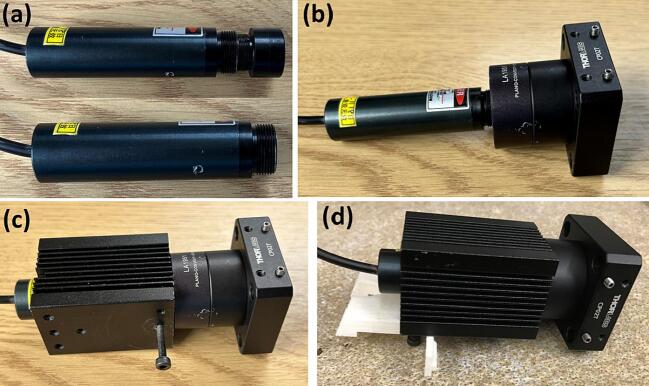
(a) 980 nm laser (240 mW) with and without the original lens; assembly of 980 nm laser diode to the (b) optics and (c) heatsink/housing; (d) attachment of the 980 nm laser to the 3D printed clip-on mount.

**Step 7:** Remove the original lens from the 980 nm laser (**Laser housing 1**), as shown in Fig. 4 (a).

**Step 8:** Attach the 980 nm laser (**Laser housing 1**) to the part assembled in **Step 6**, as shown in Fig. 4 (b).

**Step 9:** Insert the part assembled in **Step 8** into the heatsink/laser diode housing (**Laser housing 2**) and secure it using an M3 screw (**Laser housing 3**), as shown in Fig. 4 (c).

**Step 10:** Secure the part assembled in **Step 9** to the 3D printed mount (**Step 1**) using an M3 (**Laser housing 3**), as shown in Fig. 4 (d).

*Attachment of laser to the FFF printer:*

**Fig. 5:**
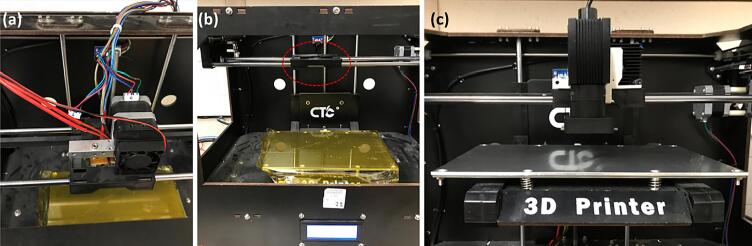
(a-b) Removal of extruders from CTC Bizer; (c) installation of the 980 nm laser/housing via a 3D printed mechanical mount.

The FFF printer (CTC Bizer) used in this article had two damaged extruders, therefore they were both removed, alongside the cooling fans, as shown in Fig. 5 (a-b). This is, however, not necessary, if the extruders are working properly.

**Step 11:** Attach the laser/housing/mount assembled in **Step 10** to the front of the extruder holder compartment via the clip-on mechanism of the 3D printed mechanical mount, as shown in Fig. 5 (c). The heatsink and the motor of the original right extruder, were left in place in order to touch the limit switch during homing.

*Laser control electronics assembly:*

The CTC Bizer printer has the same open-source mainboard (Mighty Board, version E) as MakerBot Replicator and its clones [40]. The Mighty Board has an ‘EXTRA’ FET pin, which is usually used to control a fan for ‘active cooling’, but can also be used to control a laser driver.

**Fig. 6:**
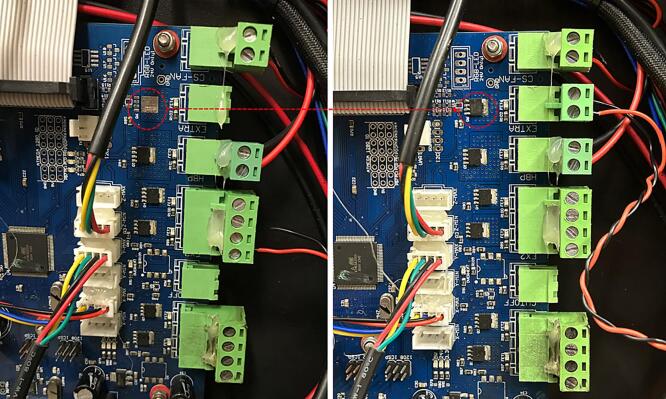
Installation of an extra MOSFET onto the Mighty Board of the CTC Bizer 3D printer.

**Step 12:** In case of the CTC Bizer, its Mighty Board is missing a metal–oxide–semiconductor field-effect transistor (MOSFET) in the ‘EXTRA’ pin, therefore an N-Channel MOSFET needs to be soldered to the pad highlighted in red circle, as shown in Fig. 6.

**Fig. 7:**
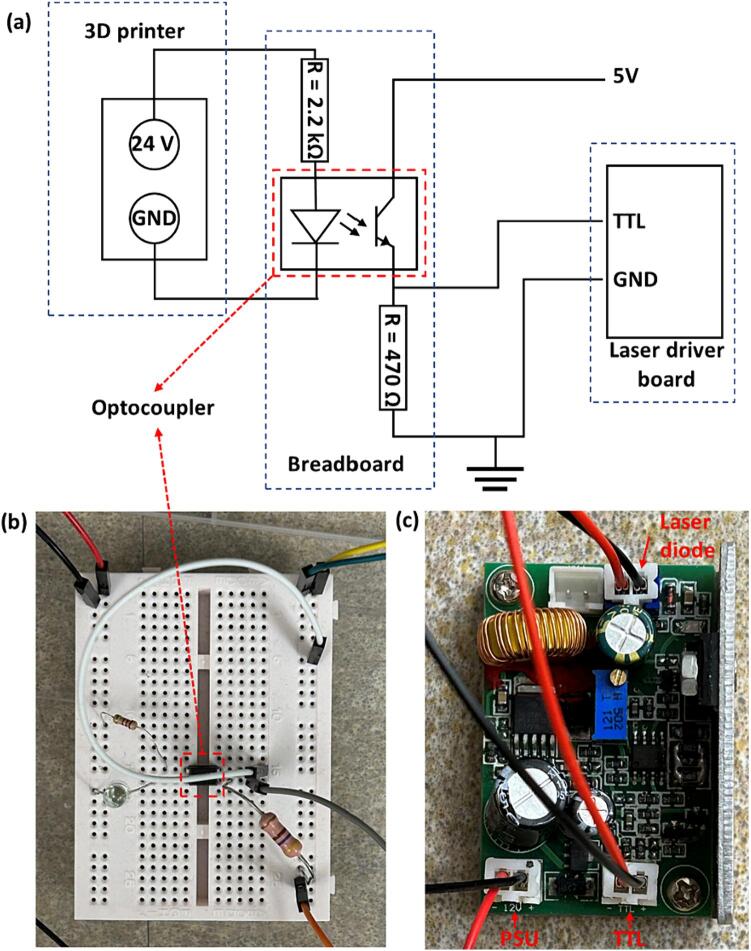
(a) Schematics of laser control circuit used in the modified FFF printer equipped with a 980 nm diode laser; (b) breadboard with an optocoupler component; (c) laser driver board.

**Step 13:** To control the laser, transistor-transistor-logic (TTL) control unit on the laser driver board (**Laser control 2**) can be used. Since the ‘EXTRA’ FET has a 24 V output, not 5 V required for TTL control in the laser driver, an external circuit shown in Fig. 7 (b,c), should be assembled on a breadboard (**Laser control 3**) using **Laser control 4**–**7** components in the Bills of Materials table. The optocoupler (**Laser control 4**) is used to separate the 3D printer’s output signal from the laser driver’s (**Laser control 2**) input signal. This circuit can also be used with FFF printers that have a lower output (e.g. 12 V or 5 V) to control their fans. In this case, the 2.2 kΩ resistor (**Laser control 4**) needs to be replaced with another one to take into account the reduced voltage.

**Step 14:** Connect the circuit built in **Step 13** to the ‘EXTRA’ FET pins (Fig. 6) of the 3D printer, using the red and black wires (**Laser control 8,9**).

**Step 15:** Connect the circuit built in **Step 13** to the TTL pin of the laser driver board (**Laser control 2**), as shown in Fig. 7 (c).

**Step 16:** Connect the laser driver board (**Laser control 2**) to the power supply unit (PSU).

**Step 17:** Connect the laser driver board (**Laser control 2**) to the laser diode attached to the 3D printer in **Step 11**.

**Step 18:** Connect the circuit built in **Step 13** to the 5 V output AC to DC adapter, which should be plugged in to the mains. This is for providing voltage for the TTL control unit.

## 6 Operation instructions

*Initial Software Installation and Setup:*

**Fig. 8:**
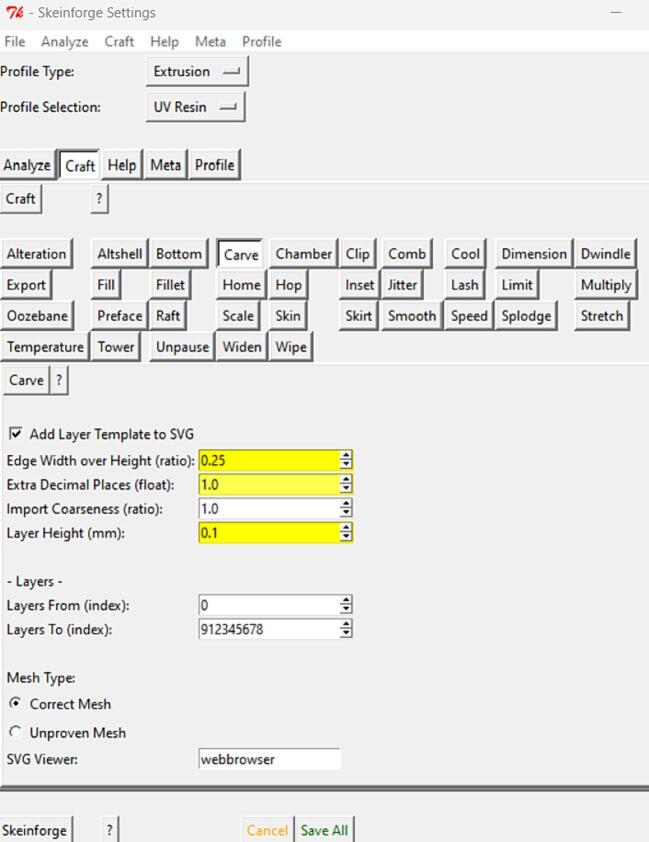
Skeiforge Settings window.

• Download and install ReplicatorG software onto your computer from replicat.org website [38].
• Download “uvlprinter.xml” and “UV3D.1mm.zip” files from the Instructables by Andy Rosen [27].
• Move the “uvlprinter.xml” file into the machines directory inside the ReplicatorG folder.
• Open ReplicatorG software and click on the “Machine” menu. Select “Machine Type (Driver)” and choose “The Replicator Dual”. Next, select the serial port connected to your 3D printer.
• Under the “GCode” menu, select “GCode Generator” and choose “Skeinforge (50)”.
• Click on the “File” menu, then “Examples” and select any of the example STL files. Under the “Gcode” menu click on “Edit Slicing Profiles”. Then duplicate the profile, rename it to the name of your choosing, and open this profile's folder by clicking on “Locate” option. Replace all content with those from the “UV3D.1 mm.zip” file.
• Open the “Edit Slicing Profiles” window again and click on your newly created profile and then press “Edit”, which will open the Skeiforge Settings window for this profile, as shown in Fig. 8. There you can change various printing settings (printing speed, layer thickness, the distance between each laser path for the shell and fill, etc.).

*Converting a 3D design into a G-code script:*

**Fig. 9:**
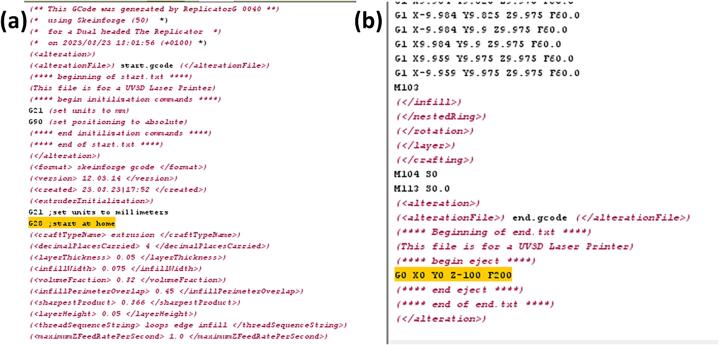
Remove lines (a) “G28;start at home” at the start and (b) “G0 X0 Y0 Z-100 F200” at the end of the generated GCode script.
• Save your edited G-code, connect your computer to the 3D printer using a USB cable, and click “Connect”.
• Ensure the power supply unit (PSU) is turned on and the 5 V output AC to DC adapter is plugged in.
• The laser power can be adjusted by changing the current in the PSU. Make sure that the maximum allowable current value specified by the supplier of the laser diode is not exceeded, to avoid damage to the laser diode.

• Open your CAD design file (must be in the STL format) in ReplicatorG.
• Navigate to the “GCode” menu and select “Generate”. In the opened window, choose the new slicing profile you have created in the previous steps, then click on “Generate Gcode”.
• Once the G-code script is generated, it can be viewed and edited in the G-code section. Note: speed is represented in the G-code script in mm/min units, for instance F60.0 is 60 mm/min or 1 mm/s.
• In the generated G-code script, replace all instances of “M101″ with ”M126″ (laser on command), and “M103″ with ”M127″ (laser off command). This can be done using Ctrl + F to find the commands and clicking on “Replace All”.
• Remove lines “*G28;start at home*” at the start and “*G0 X0 Y0 Z-100 F200*” at the end of the generated G-code script, as shown in Fig. 9, in order to prevent Z-axis homing and potential damage to the laser/mount.

*Adjusting the Z-height before the print:*

• Before starting the print, firstly manually align the laser's focal length with the platform by moving the Z-axis. This is done via “Utilities” and “Jog Mode” of the 3D printer screen interface.
• Then micro-adjust Z platform to make sure that it is at the focal length away from the focusing lens of the laser, by using ReplicatorG commands, for example: “G21 G90 G1 Z0.8 F500.0″, where the “Z” value represents movement in mm, and a positive value indicates the build platform moving downwards and negative value upwards.

*Preparing and starting the 3D print:*

• Prepare the resin formation, which contains upconverting particles, for instance the formulations described in References [21], [22].
• Place the resin formulation in a suitable container (for example plastic or glass beaker) and make sure that the walls of the container are shorter than the focal length of the focusing length. Place the container directly below the laser.
• Lastly, open the G-code script generated earlier from your STL design and click the “Build” button in the ReplicatorG software to begin the printing process.

*Safety Considerations:*

• Ensure you are in a well-ventilated area when using the 3D printer.
• When the laser is on, always wear the NIR laser protective glasses, for example the ones in Reference [41].
• Do not attempt to touch moving parts during operation.

## 7 Validation and characterization

**Fig. 10:**
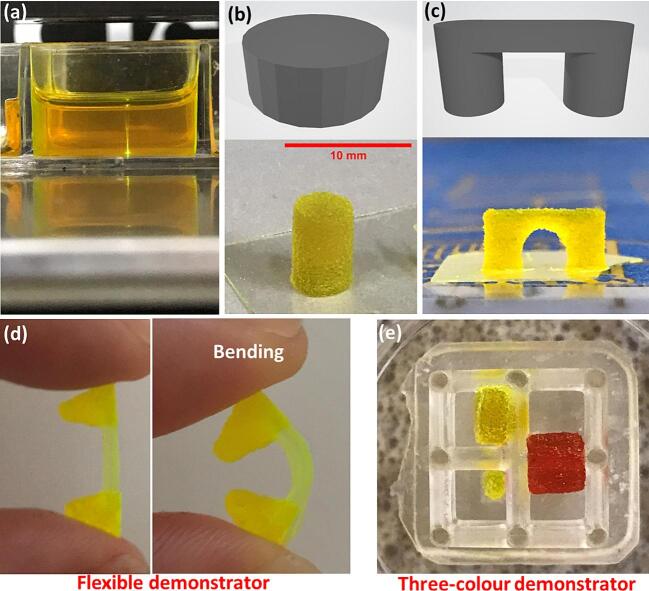
(a) The modified FFF printer in operation; (b) ‘cylinder’ design and 3D printed sample; (c) ‘bridge’ design and 3D printed sample; (d) flexible/rigid sample; (e) multi-color parts printed inside of a LCD printed structure.

The modified FFF printer was used to 3D print using the NIR (980 nm) laser via selective crosslinking in photopolymer resin at an intended depth as per the requirement, as shown in Fig. 10 (a). This is achieved due to the addition of upconverting NaYF_4_: (20 %)Yb^3+^, (3 %)Tm^3+^ phosphor into the resin formulation, which upon sequential absorption of 980 nm photons, emits higher-energy photons in the UV and visible light range [21]. The energy dose needs to be carefully optimized in order to be able to provide enough energy to cure the the resin, however not too much in order to avoid curing outside of the focal point of the laser. For instance, as Fig. 10 (b) shows, at the laser power density (P.D.) of 3372 W cm^−2^ and printing speed of 1 mm s^−1^ corresponding to an (E.D.) of 252 J cm^−2^, the object is significantly over-cured in the z-axis. To avoid over-curing, a P.D. of 1405 W cm^−2^ and printing speed of 1 mm s^−1^ corresponding to energy density E.D. of 70 J cm^−2^ was used to 3D print a bridge shown in Fig. 10 (c), with the area below the bridge, remaining uncured. This method was also demonstrated in fabrication of a rigid/flexible (acrylic/elastomer) sample (Fig. 10 (d)). This involved printing the flexible part (middle) with a traditional LCD printer, followed by immersing the part in the modified resin formulation and printing the rigid parts with NIR laser using the modified FFF printer presented in this work. This approach was also used to print features in different colors inside of a previously LCD printed part, as shown in Fig. 10 (e).

*Key capabilities of the technology include (but are not limited to):*

• The high penetration depth of NIR light allows selective crosslinking at various depths.
• Possibility to 3D print structures/features inside previously 3D printed parts.
• The process can be applied to a wide range of photopolymer resins (including high viscosity formulations).

*Limitations:*

• Limited resolution. This, however, could be increased by improving the optical setup, which would allow the minimization of the laser spot size (for instance, by using a lens with higher numerical aperture (NA), while preserving a long-enough working distance).
• Slow printing speed. This can be improved by using a higher power 980 nm laser, as well as improving the resin formulations for higher sensitivity (using more efficient UC phosphors, more sensitive initiators, etc).
• Manual focus adjustment before starting a print. This can be automated with the introduction of an auto-focus sensor kit commonly used with the laser engravers and cutters, and modification to the printing software.
• Further work in improving software/firmware specifically tailored to this technique is required.

## CRediT authorship contribution statement

**Adilet Zhakeyev:** Formal analysis, Investigation, Methodology, Visualization, Writing – original draft, Writing – review & editing. **Rohith Devanathan:** Writing – original draft. **Jose Marques-Hueso:** Conceptualization, Funding acquisition, Project administration, Supervision, Writing – review & editing.

## Declaration of competing interest

The authors declare that they have no known competing financial interests or personal relationships that could have appeared to influence the work reported in this paper.
